# Agent of Whirling Disease Meets Orphan Worm: Phylogenomic Analyses Firmly Place Myxozoa in Cnidaria

**DOI:** 10.1371/journal.pone.0054576

**Published:** 2013-01-30

**Authors:** Maximilian P. Nesnidal, Martin Helmkampf, Iris Bruchhaus, Mansour El-Matbouli, Bernhard Hausdorf

**Affiliations:** 1 Zoological Museum, University of Hamburg, Hamburg, Germany; 2 Bernhard Nocht Institute for Tropical Medicine, Hamburg, Germany; 3 Clinical Division of Fish Medicine, University of Veterinary Medicine, Vienna, Austria; BiK-F Biodiversity and Climate Research Center, Germany

## Abstract

Myxozoa are microscopic obligate endoparasites with complex live cycles. Representatives are *Myxobolus cerebralis*, the causative agent of whirling disease in salmonids, and the enigmatic “orphan worm” *Buddenbrockia plumatellae* parasitizing in Bryozoa. Originally, Myxozoa were classified as protists, but later several metazoan characteristics were reported. However, their phylogenetic relationships remained doubtful. Some molecular phylogenetic analyses placed them as sister group to or even within Bilateria, whereas the possession of polar capsules that are similar to nematocysts of Cnidaria and of minicollagen genes suggest a close relationship between Myxozoa and Cnidaria. EST data of *Buddenbrockia* also indicated a cnidarian origin of Myxozoa, but were not sufficient to reject a closer relationship to bilaterians. Phylogenomic analyses of new genomic sequences of *Myxobolus cerebralis* firmly place Myxozoa as sister group to Medusozoa within Cnidaria. Based on the new dataset, the alternative hypothesis that Myxozoa form a clade with Bilateria can be rejected using topology tests. Sensitivity analyses indicate that this result is not affected by long branch attraction artifacts or compositional bias.

## Introduction

Myxozoa are microscopic obligate endoparasites with complex live cycles that differ between the two subgroups Myxosporea and Malacosporea [1]–[3]. Myxosporea includes about 2,180 species, with a myxospore phase resulting in the production of myxospores in lower vertebrates, typically in fish, rarely in amphibians and reptiles, and an actinospore phase that engages in sexual reproduction and results in the production of actinospores, in annelids. Just four malacosporean species are known, all parasitizing freshwater bryozoans and some having life stages parasitizing in fish [4], [5]. The enigmatic “orphan worm” *Buddenbrockia plumatellae*, which displays a worm-like trophic stage, belongs to this group [6], [7]. Several Myxozoa are economically important pathogens such as the myxosporean *Myxobolus cerebralis*, the causative agent for whirling disease in salmonids.

Originally, Myxozoa were classified as protists [8]. Later several metazoan characteristics, namely multicellularity of some life stages, tight junctions, and collagen were detected [1], [2], [9]. Phylogenetic analyses of 18S rDNA sequences confirmed the placement of Myxozoa within Metazoa and placed them as sister group to or even within Bilateria [6], [10]–[17]. This placement was further confirmed by bilaterian-like *Hox* genes [18], [19] and by the identification of *Buddenbrockia* as a myxozoan with longitudinal muscles [6], [7].

However, all Myxozoa possess polar capsules that are similar to nematocysts of Cnidaria in ultrastructure and ontogeny and are used for host attachment [9], [20]. Based on these structures, a close relationship between Myxozoa and Cnidaria has been postulated [9], [20]. Specifically, some phylogenetic analyses of 18S rDNA sequences of myxosporeans indicated a sister group relationship between Myxozoa and the parasitic cnidarian *Polypodium* [9], [21], [22]. The name Endocnidozoa has been established for this clade [15]. A clade including Myxozoa and Cnidaria has also been supported by the presence of a minicollagen gene in the malacosporean *Tetracapsuloides bryosalmonae* [23]. Minicollagens are cnidarian-specific constituents of nematocyst walls. Furthermore, phylogenetic analyses of EST data of the malacosporean *Buddenbrockia* [19] placed Myxozoa as sister group of Medusozoa within Cnidaria. However, a topology test could not reject a bilaterian origin for *Buddenbrockia* [19].

Molecular phylogenetic analyses of the relationships of Myxozoa are complicated by their extremely high substitution rates, possibly resulting in long-branch attraction artifacts [11], [12], [14]–[17], [22]. Evans et al. [17] demonstrated that there are conflicting signals in the phylogenomic data of *Buddenbrockia* and that a removal of just a few sites from this dataset changes the placement of Myxozoa from within Cnidaria to the alternative hypothesis at the base of Bilateria in the maximum likelihood tree.

We sequenced a part of the genome of *Myxobolus cerebralis* (Myxosporea) and compiled a large dataset for phylogenomic analyses to more robustly resolve the relationships of Myxozoa. We constructed a phylogenetic tree based on a dataset including 128 genes, investigated in how far the tree may be affected by long branch attraction artifacts or compositional bias and tested alternative hypotheses concerning the position of Myxozoa with a topology test.

## Methods

### Sampling and DNA Extraction

Triactinomyxon spores of *Myxobolus cerebralis* were concentrated from the water over a highly infected *Tubifex tubifex* laboratory culture as described by El-Matbouli & Soliman [24]. The triactinomyxon spores were preserved in ethanol and cleaned with Pasteur pipettes. The samples were homogenized by passing through a blunt 20-gauge needle. Genomic DNA was extracted using the QIAamp DNA Mini kit (Qiagen, Hilden, Germany) according to the manufacturer’s instructions with the exception that the samples were incubated with proteinase K over night at 37°C.

### Genomic Sequencing

Whole-genome shotgun data were obtained by running one PicoTiterPlate on the GS FLX Titanium platform (454 Life Sciences, Branford, CT). The library for 454 sequencing was prepared with the GS FLX Titanium General Library Preparation Kit (Roche, Mannheim, Germany), immobilized on beads and clonally amplified using the GS FLX Titanium LV emPCR Kit (Lib-L) (Roche, Mannheim, Germany). The library was then sequenced with the GS FLX Titanium Sequencing Kit XLR70 (Roche, Mannheim, Germany) and the GS FLX Titanium PicoTiterPlate Kit (Roche, Mannheim, Germany). All kits were used according to the manufacturer’s protocol. Sequence assembly was performed using MIRA version 3 rc4 (development version) [25].

The genomic sequences have been deposited in the Sequence Read Archive (http://www.ncbi.nlm.nih.gov/Traces/sra/) under accession number SRA062243.

### Data Assembly

We used Exonerate version 2.2 [26] to extract orthologs of the 128 protein genes selected by Philippe et al. [27] from the genomic data of *Myxobolus cerebralis* and the EST data of *Buddenbrockia plumatella* [19] with a query set of these protein genes from *Nematostella vectensis*. The sequences were translated using EMBOSS Transeq [28]. The orthology relationships of the *Myxobolus* sequences were confirmed by constructing gene trees in case of doubt. We added the sequences of *M. cerebralis* and *B. plumatella* to a previously assembled phylogenomic dataset with intensively sampled basal metazoans including nine sponges, nine cnidarians, three ctenophores, one placozoan, and 22 slowly evolving bilaterian taxa [27].

### Alignment, Alignment Masking and Gene Selection

The amino acid sequences of the individual ortholog groups were aligned with MAFFT using the most sensitive option L-INS-i [29], [30]. To increase the signal-to-noise ratio sections with only random sequence similarity were identified with ALISCORE version 1.0 [31], [32] and subsequently excluded with ALICUT (http://www.utilities.zfmk.de). This step removed 5.482 of originally 38.415 amino acid positions. All masked alignments were concatenated to a superalignment comprising 32,933 amino acid positions, which has been deposited at TreeBASE (http://www.treebase.org, accession number S13690). The protein genes available for the individual taxa are listed in Table S1. Overall 30% of the amino acids in the matrix are missing.

### Phylogenetic Analyses

We performed maximum likelihood analyses using a parallel Pthreads-based version of RAxML version 7.3.0 [33], [34] with the LG+F+G model [35]. Based on the complete alignment, we computed 10 maximum likelihood trees using 10 distinct randomized maximum parsimony trees and choose the tree with the highest likelihood. Confidence values for edges of the maximum likelihood tree were computed by rapid bootstrapping [36] (100 replications).

We performed a Bayesian inference analysis with the CAT model that adjusts for site-specific amino acid frequencies [37] as implemented in PhyloBayes version 3.3b (http://megasun.bch.umontreal.ca/People/lartillot/www/index.htm). Four independent chains were run for 30,000 points. 5,000 points were discarded as burn-in. The largest discrepancy observed across all bipartitions (maxdiff) is 0.19. Taking every 10th sampled tree, a 50%-majority rule consensus tree was computed using all chains.

To test predefined phylogenetic hypotheses, we calculated the maximum-likelihood tree for a specified hypothesis by resolving multifurcations in a constrained tree with RAxML. Next, we computed per-site log likelihood scores for the global and the constrained maximum-likelihood trees with RAxML and performed an approximately unbiased test [38] using CONSEL [39] to investigate whether the alternative hypotheses can be rejected.

### Influence of Compositional Heterogeneity and Rate Heterogeneity Among Lineages on the Phylogenetic Analyses

Effects of compositional heterogeneity between lineages on the phylogenetic analyses can be mitigated by the exclusion of data partitions that show compositional heterogeneity between lineages [40], [41]. To facilitate the identification of such partitions, we scored the compositional heterogeneity of all single protein alignments using relative composition variability, the average variability in composition between taxa [42], but using frequencies instead of absolute numbers [43]. We adapted the formula of relative composition frequency variability (*RCFV*) to amino acids instead of nucleotides:
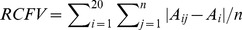
where *A_ij_* is the frequency of amino acid *i* in taxon *j*, and *A_i_* is the average frequency of amino acid *i* across the *n* taxa. Constant sites were excluded from the *RCFV* calculations.

Similarly, effects of rate heterogeneity between lineages can be mitigated by the exclusion of fast evolving character subsets [44], [45]. We ranked all individual proteins according to their relative substitution rates estimated as the sum of all pair-wise maximum likelihood distances between the sequences of the 15 species from which sequences of all used proteins were available (Table S1). The ML distances were computed with RAxML and the LG+G+F model.

To evaluate the influence of compositional heterogeneity and rate heterogeneity among lineages on the phylogenetic analyses, we constructed 8 additional datasets by excluding 0, 10 and 33% of the most heterogeneous protein sequences and/or 0, 10 and 33% of the fastest evolving proteins.

## Results and Discussion

**Figure 1:**
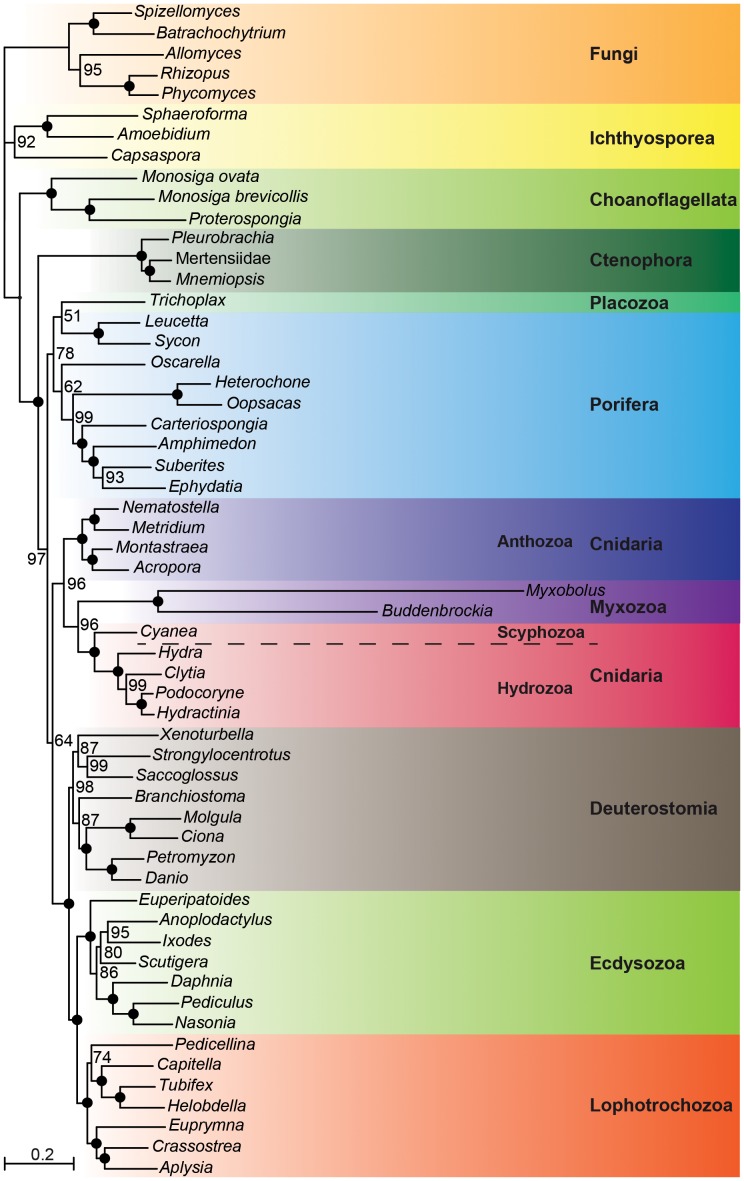
Maximum likelihood tree calculated with the LG+G+F model based on 32,933 amino acid positions derived from 128 proteins of 57 taxa. Bootstrap values larger than 50% are shown to the right of the nodes; 100% bootstrap values are indicated by black circles.

Seventy-nine of the 128 genes of the dataset compiled by Philippe et al. [27] were extracted from the new genomic data of the myxosporean *Myxobolus cerebralis*. Moreover, 60 of these genes were extracted from EST data of the malacosporean *Buddenbrockia plumatella* [19]. A maximum likelihood analysis of the complete dataset including 32,933 amino acid positions considering the two myxozoan, 44 additional metazoan and 11 outgroup taxa revealed a sister group relationship of *Myxobolus* and *Buddenbrockia* (100% bootstrap support; Fig. 1). The two myxozoans form the sister group of Medusozoa within Cnidaria (96% bootstrap support). The two myxozoans also formed a clade with Medusozoa in a Bayesian inference analysis with the CAT model (Figure S1). However, this clade is only very weakly supported (0.64 posterior probability) and the relationships within this clade are unresolved. It has been shown that phylogenetic analyses with CAT model may be misled by heterogeneity of substitution profiles, the equilibrium frequencies over the twenty amino acids at the individual sites, over time [46]. Actually, such a systematic error affected the reconstruction of the phylogenetic relationships among diploblasts based on mitochondrial protein sequences [46] and it may also be the cause of the weak support for the relationships of myxozoans within diploblasts in our analysis.

Evans et al. [17] suggested that the substantial amount of missing data may have affected the phylogenetic placement of Myxozoa in the phylogenomic study of Jiménez-Guri et al. [19] and that the effects of missing data should further be explored. To test the potential influence of missing data on the phylogenetic placement of Myxozoa, we produced a trimmed dataset by excluding all positions from the amino acid alignment for which no sequence information from at least one of the myxozoans was available. The maximum likelihood analysis of the resulting dataset including 18,273 amino acid positions confirmed the sister group relationship between Myxozoa and Medusozoa (97% bootstrap support; Figure S2).

Our analyses corroborate the placement of Myxozoa in Cnidaria as has been suggested based on the possession of polar capsules that are similar to nematocysts of Cnidaria [9], [20], the presence of a minicollagen gene otherwise known only from Cnidaria [23] and the phylogenetic analyses of 18S rDNA sequences of myxosporeans [9], [22] and EST data of the malacosporean *Buddenbrockia* [19]. However, previous sequence datasets were not sufficient to reject the alternative hypothesis that Myxozoa (or Endocnidozoa) are the sister group of Bilateria or are even a branch within Bilateria [6], [10]–[17]. With the newly compiled sequence data, this hypothesis could be significantly rejected using the approximately unbiased test (Table 1). Thus, the new genomic data unambiguously support the evolution of the parasitic Myxozoa from Cnidaria.

**Table 1:** Results of the approximately unbiased test.

phylogenetic hypothesis	references claiming the hypothesis	likelihood	Δ likelihood^a^	AU^b^
ML tree		−985,545	0	0.998
Myxozoa form a clade with Bilateria	6, 10–17	−985,636	91	0.002*
Myxozoa form a clade with Hydrozoa	9, 22	−985,647	102	0.000*

a Δ Likelihood: differences between the likelihood of a constrained tree and the maximum likelihood tree.

b AU: approximately unbiased test (*p*-values). Values for topologies significantly rejected at the 0.05 level are indicated by an asterisk.

There are also different hypotheses concerning the relationships of Myxozoa within Cnidaria. Some authors [9], [21], [22] concluded that Myxozoa are the sister taxon of *Polypodium*, which has been classified in the hydrozoan clade Narcomudusae (Trachylina) at that time. However, Evans et al. [16] suggested that *Polypodium* is either the sister taxon of Hydrozoa or part of the hydrozoan clade Leptothecata (Hydroidolina). In any case, this would mean that Myxozoa form a clade with Hydrozoa. In contrast, Myxozoa are the sister taxon of Medusozoa in the phylogenomic analysis of Jiménez-Guri et al. [19]. The latter hypothesis is supported by our phylogenetic analyses (Fig. 1). If we constrain the monophyly of Myxozoa+Hydrozoa, Myxozoa are placed as sister group of all Hydrozoa in the constrained maximum likelihood tree. They do not group with *Clytia*, a representative of the Leptothecata in our dataset, as we would expect if Myxozoa and *Polypodium* are part of Leptothecata. Rather, the topology test with our dataset permits to reject the hypothesis that Myxozoa form a clade with Hydrozoa (Table 1).

The topology tests have shown that the placement of Myxozoa as the sister taxon of Medusozoa within Cnidaria cannot be explained by random errors. However, systematic errors resulting from model violations like heterogeneity in substitution rates or amino acid composition among lineages are not considered in topology tests and bootstrap analyses and can confound accurate tree reconstruction [20], [44], [47], [48]. Just as *Buddenbrockia plumatella*, *Myxobolus* is characterized by a very high substitution rate. Whereas the average distance from the base of the maximum likelihood tree (Fig. 1) to the terminal nodes is 0.466, the distance of *Myxobolus* is 1.521. The average pairwise amino acid distance calculated with the LG+F+G model in the complete data set is 0.492, the average pairwise distance between *Myxobolus* and other taxa is 1.111. The highest pairwise distance has been recorded between *Myxobolus* and the mertensiid ctenophore (1.286). In fact, it has been supposed that the placement of myxozoans, might be affected by long branch attraction [11], [12], [14]–[17].

We therefore assessed the influence of proteins with varying evolutionary rates and amino acid compositions among lineages on the inferred phylogenetic relationships of the myxozoans via a step-wise exclusion analysis. We removed the 10% or 33% fastest evolving proteins and/or the 10% or 33% proteins with the highest relative composition variability from the dataset and repeated the phylogenetic reconstruction. The monophyly of Myxozoa was confirmed in all of the resulting 8 trees with 100% bootstrap support (Figures S3–S10). The support for the monophyly of Cnidaria inclusive Myxozoa and of Myxozoa+Medusozoa was high (88–99%) in all of these analyses (Table 2; Figures S3–S10). Thus, these sensitivity analyses corroborate the robustness of the placement of Myxozoa as sister group to Medusozoa within Cnidaria.

**Table 2:** Sensitivity of the phylogenetic analysis to rate heterogeneity and compositional heterogeneity among lineages.

Approach	no exclusion of heterogeneous proteins	exclusion of 10% most heterogeneous proteins	exclusion of 33% most heterogeneous proteins
no exclusion of fast proteins	96	98	99
	*96*	*98*	*99*
exclusion of 10% fastest proteins	98	99	96
	*98*	*99*	*96*
exclusion of 33% fastest proteins	90	88	93
	*90*	*88*	*94*

Bootstrap support values for the monophyly of Cnidaria inclusive Myxozoa (upper values) and of Myxozoa+Medusozoa (lower values in italic).

The next step will be the generation of genomic data for *Polypodium* so that the hypothesis that this parasitic cnidarian represents the sister group of Myxozoa [9], [21], [22] can be tested in phylogenomic analyses.

### Conclusion

The phylogenetic placement of Myxozoa within Cnidaria might have important implications for parasitologists and veterinaries seeking to develop strategies for avoiding infection with these economically important parasites and combating the resulting diseases. Our results expose the evolutionary plasticity of the cnidarian bauplan, which gave rise to obligatory parasitic organisms reminiscent of protozoa or bilaterian worms from free-living, radially symmetrical origins. The loss of eumetazoan apomorphies like epithelial tissue layers, gut, nerve and sensory cells, shows that Myxozoa are a primary example for the extreme reduction of complexity that comes with the evolution of a parasitic lifestyle. More detailed analyses of myxozoan genomes in the future might further strengthen our understanding of metazoan evolution by revealing the genetic underpinnings that drive these profound changes during myxozoan development.

## Supporting Information

Figure S1 **Bayesian inference reconstructions with the CAT model based on 32,933 amino acid positions derived from 128 proteins of 57 taxa.** Bayesian posterior probabilities are shown to the right of the nodes.
(PDF)

Figure S2 **Maximum likelihood tree calculated with the LG+G+F model based on 18,273 amino acid positions derived from 128 proteins (after excluding all positions for which no sequence information is available from myxozoans).** Bootstrap values larger than 50% are shown to the right of the nodes.
(PDF)

Figure S3 **Maximum likelihood tree calculated with the LG+G+F model based on 31,488 amino acid positions derived from 115 proteins (after excluding the 10% most heterogeneous proteins).** Bootstrap values larger than 50% are shown to the right of the nodes.
(PDF)

Figure S4 **Maximum likelihood tree calculated with the LG+G+F model based on 26,974 amino acid positions derived from 85 proteins (after excluding the 33% most heterogeneous proteins).** Bootstrap values larger than 50% are shown to the right of the nodes.
(PDF)

Figure S5 **Maximum likelihood tree calculated with the LG+G+F model based on 30,565 amino acid positions derived from 115 proteins (after excluding the 10% fastest evolving proteins).** Bootstrap values larger than 50% are shown to the right of the nodes.
(PDF)

Figure S6 **Maximum likelihood tree calculated with the LG+G+F model based on 29,712 amino acid positions derived from 106 proteins (after excluding the 10% fastest evolving proteins and the 10% most heterogeneous proteins).** Bootstrap values larger than 50% are shown to the right of the nodes.
(PDF)

Figure S7 **Maximum likelihood tree calculated with the LG+G+F model based on 26,760 amino acid positions derived from 84 proteins (after excluding the 10% fastest evolving proteins and the 33% most heterogeneous proteins).** Bootstrap values larger than 50% are shown to the right of the nodes.
(PDF)

Figure S8 **Maximum likelihood tree calculated with the LG+G+F model based on 23,553 amino acid positions derived from 85 proteins (after excluding the 33% fastest evolving proteins).** Bootstrap values larger than 50% are shown to the right of the nodes.
(PDF)

Figure S9 **Maximum likelihood tree calculated with the LG+G+F model based on 23,289 amino acid positions derived from 81 proteins (after excluding the 33% fastest evolving proteins and the 10% most heterogeneous proteins).** Bootstrap values larger than 50% are shown to the right of the nodes.
(PDF)

Figure S10 **Maximum likelihood tree calculated with the LG+G+F model based on 21,643 amino acid positions derived from 67 proteins (after excluding the 33% fastest evolving proteins and the 33% most heterogeneous proteins).** Bootstrap values larger than 50% are shown to the right of the nodes.
(PDF)

Table S1 **Protein genes available from the studied taxa.**
(XLSX)
