# Supplementary material for: Agent of Whirling Disease Meets Orphan Worm: Phylogenomic Analyses Firmly Place Myxozoa in Cnidaria
Source: PLoS One. 2013 Jan 30;8(1):e54576. doi: 10.1371/journal.pone.0054576 (PMC3559788; doi:10.1371/journal.pone.0054576)

Figure S1. Bayesian inference reconstruction with the CAT model based on 32,933 amino acid positions derived from 128 proteins of 57 taxa. Bayesian posterior probabilities are shown to the right of the nodes.

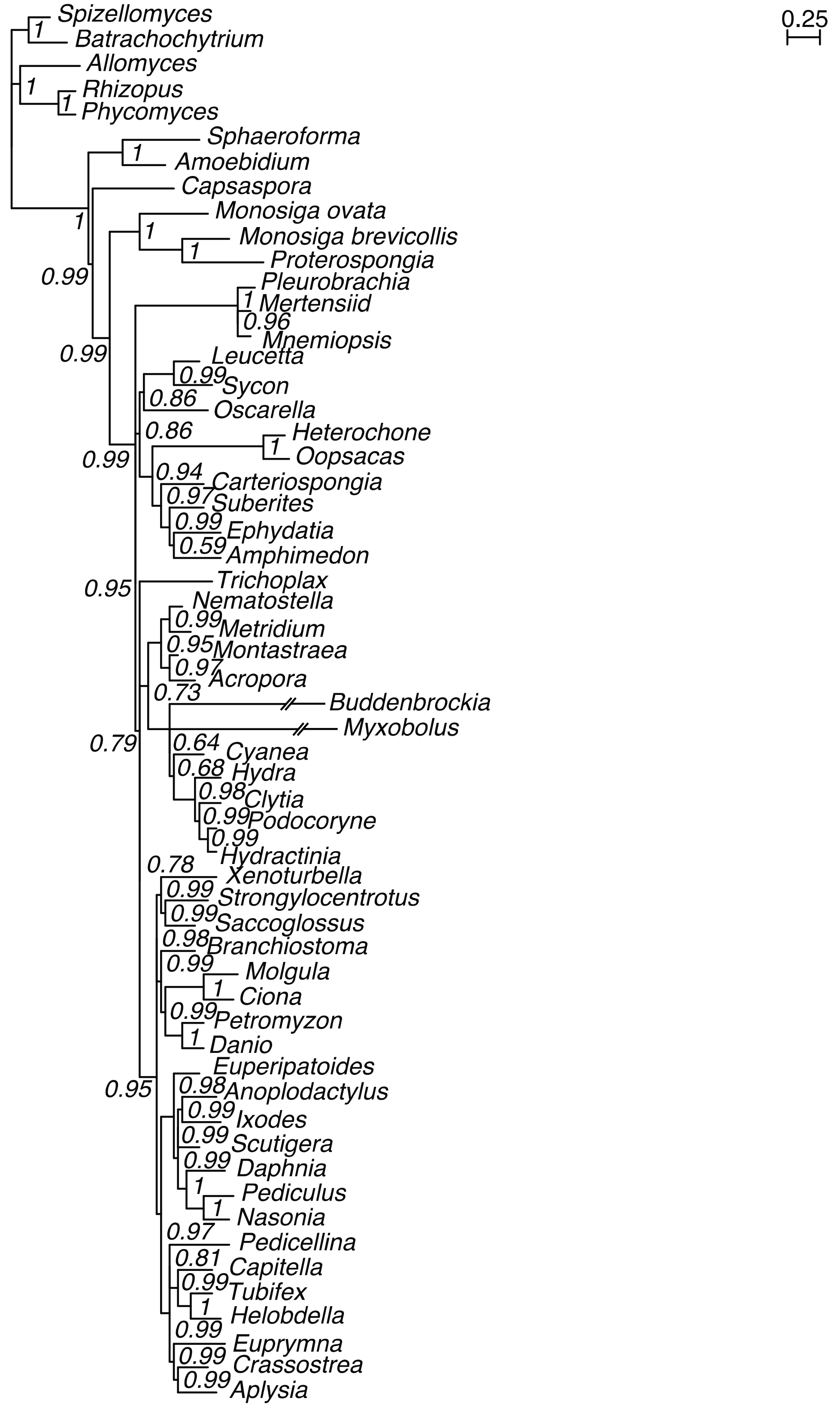

Supplement: Figure S1 — Bayesian inference reconstructions with the CAT model based on 32,933 amino acid positions derived from 128 proteins of 57 taxa. Bayesian posterior probabilities are shown to the right of the nodes. (PDF) [file pone.0054576.s001.pdf]
