# Supplementary material for: Agent of Whirling Disease Meets Orphan Worm: Phylogenomic Analyses Firmly Place Myxozoa in Cnidaria
Source: PLoS One. 2013 Jan 30;8(1):e54576. doi: 10.1371/journal.pone.0054576 (PMC3559788; doi:10.1371/journal.pone.0054576)

Figure S9. Maximum likelihood tree calculated with the LG+G+F model based on 23,289 amino acid positions derived from 81 proteins (after excluding the 33% fastest evolving proteins and the 10% most heterogeneous proteins). Bootstrap values larger than 50% are shown to the right of the nodes.

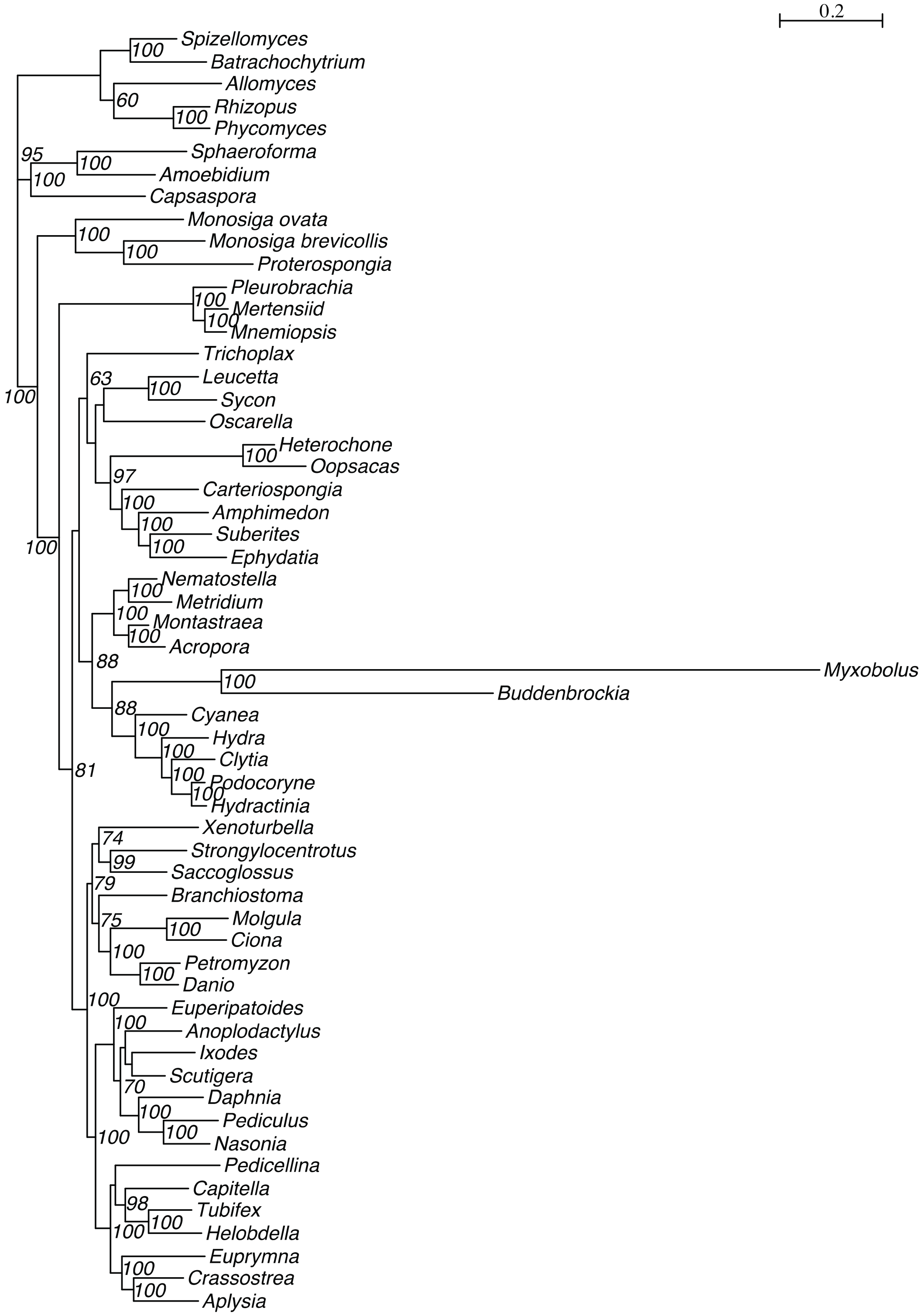

Supplement: Figure S9 — Maximum likelihood tree calculated with the LG+G+F model based on 23,289 amino acid positions derived from 81 proteins (after excluding the 33% fastest evolving proteins and the 10% most heterogeneous proteins). Bootstrap values larger than 50% are shown to the right of the nodes. (PDF) [file pone.0054576.s009.pdf]
